# Identification of Pyroptosis-Related Gene Signatures and Construction of the Risk Model to Predict BCR in Prostate Cancer

**DOI:** 10.3389/fmolb.2022.850758

**Published:** 2022-06-23

**Authors:** Miaomiao Wang, Haoran Xia, Qiuxia Yan, Wen Liu, Ming Liu, Xuan Wang

**Affiliations:** ^1^ Department of Urology, Beijing Hospital, National Center of Gerontology, Institute of Geriatric Medicine, Chinese Academy of Medical Sciences, Beijing, China; ^2^ Graduate School of Peking Union Medical College, Chinese Academy of Medical Sciences, Beijing, China; ^3^ Peking University Fifth School of Clinical Medicine, Beijing, China

**Keywords:** prostate cancer, pyroptosis, TME, TMB, prognosis, BCR

## Abstract

Prostate cancer is one of the most common malignant tumors in men. Pyroptosis is related to tumor immune infiltration and tumor microenvironment (TME) and has been confirmed to be related to the progression of a variety of tumors. However, the relationship between prostate cancer and pyroptosis, as well as TME and tumor immune infiltration, has not been discussed yet. We obtained and combined the RNA-seq data of prostate cancer from TCGA and GEO databases, analyzed the differential expression of pyroptosis-related genes (PRGs), and divided them into two groups according to the PRG expression level. The relationship between pyroptosis subtypes and the TME of prostate cancer was further verified, and the differential expression genes (DEGs) in the two subtypes were identified. The relationship between the DEGs and clinicopathology was explored and KEGG and GO enrichment analysis was conducted; it was found that most DEGs were enriched in immune-related pathways. Then, we randomly divided datasets into training and testing sets, performed the LASSO and multicox progression analysis, selected eight genes as prognostic signatures and used the eight genes, calculated the risk score, and then separated the entire cohort into high- and low-risk groups. The prognosis between two groups and the 1-, 3-, and 5-year ROC curves of biochemical relapse (BCR) were verified in training, testing, and the entire cohort, respectively. The TME, CSC index, mutation, and drug susceptibility were also discussed.

## Introduction

Prostate cancer is the most common urology system malignancy tumor and the leading death cause in men worldwide (Bray et al., 2018). In recent years, with the development of the economy and the extension of the life span of the population, the incidence of prostate cancer in China has gradually increased, especially among elderly men (Ha Chung et al., 2019). For the therapeutic strategy, we mostly choose radical treatment, endocrine therapy, and other treatment methods based on the patient’s disease progression. However, after radical treatment, about one-fourth to one-half of patients still had PSA increase (Van den Broeck et al., 2019), namely biochemical relapse (BCR), which is a precursor of local recurrence or distant metastasis of prostate cancer. Biochemical recurrence is a key node in the progression of prostate cancer; after this event, the patients must be treated with salvage treatments and the options for treatment are limited. Under this situation, finding a new therapeutic target is needed to improve the tumor treatment efficiency and prolong the biochemical relapse-free survival of patients.

Pyroptosis is a type of programmed cell inflammatory necrosis (Cookson and Brennan, 2001) and a type of programmed cell death (Kovacs and Miao, 2017; Fang et al., 2020). It plays a key role in the body’s anti-infection (Jorgensen et al., 2017) and immune defense mechanism (Xu et al., 2018). Inflammation may be one of the causes of prostate cancer (Nakai and Nonomura, 2013). Pyroptosis is related to the inflammatory response of cells, and it participates in cell swelling and rupture, lysis of and the release of inflammatory factors such as IL-8, causing a strong inflammatory response. When cells were stimulated by certain factors, inflammatory caspase-1 was activated and began to cleave the Gasdermin (GSDM) protein family; then, the N-terminal domain was released to recognize the cell membrane and formed a certain tunnel leading to cell swelling, rupture, and induced cell death (Zeng et al., 2019). It also recruited immune cells to enhance the inflammatory response and led to inflammatory cell death. Recent studies have shown that pyroptosis genes play a dual role in cancer cell progression and treatment. Inflammation may lead to improper repair of the body and is related to the occurrence and progression of tumors (Karki and Kanneganti, 2019). On the other hand, the induction of pyroptosis-related genes in tumors will also inhibit carcinoma progression (Xia et al., 2019).

This article focuses on the relationship between PRGs and tumor immune infiltration during the biochemical relapse of prostate cancer in order to find relevant prognostic signatures to score the BCR risk of prostate cancer and better guide the clinical applications in the future.

## Materials and Methods

### Datasets

We downloaded the RNA-seq (fragments per kilobase million, FPKM) and clinical data of prostate cancer from TCGA (https://portal.gdc.cancer.gov) on December 6, 2021, which contained 489 prostate tumor samples and 49 adjacent-normal samples. We also downloaded the RNA-seq and clinical information including clinical T stage, G lesson score, BCR time, and BCR status of prostate cancer from GEO (https://www.ncbi.nlm.nih.gov/ geo/, ID: GSE70768, GSE11698). Then, we transformed the FPKM values of TCGA-PRAD data into transcripts per kilobase million (TPM). We also obtained the CNV (copy number various) data of prostate cancer from the USUC Xena (https://xena.ucsc.edu). All the prostate cancer mRNA expression data of 489 TCGA tumors and two GEO datasets were merged into an entire cohort and eliminated the batch effects *via* applying the “Combat” algorithm. We excluded the patients without the biochemical recurrence data; finally, a total of 1,026 prostate cancer patients were enrolled in the subsequent analyses.

### Consensus Clustering Analysis of Pyroptosis-Related Genes in Patients

Fifty-two PRGs were collected from previous studies and the MSigDB Team (REACTOME_PYROPTOSIS) (http://www.broad.mit.edu/gsea/msigdb/). According to the PRG expression, we used “ConsensusClusterPlus” packages for consensus clustering analysis and divided all the data into two different subtypes. The hallmark gene set (c2.cp.kegg. v7.2) was employed for the gene set variation analysis (GSVA).

### Identification of DEGs and the Function Annotation Analysis

The r package “limma” was performed to identify the DEGs between the two subtypes PRGs (fold change > |2|, *p* < 0.05). To further discuss the potential function of PRGs, the “Clusterprofiler” package was employed to perform KEGG and GO enrichment analysis.

### Construction and Verification of the Prognostic Model of Pyroptosis-Related Genes

To assess the prognostic value of PRGs, we utilized a univariate COX regression analysis to assess the DEG associated with biochemical recurrence. The patients were grouped into gene clusters A and B for subsequent analysis. All of the patients were randomly divided into a training set (*n* = 467) and a testing set (*n* = 467) in a 1:1 ratio, and the training set was used to construct the risk score associated with PRGs. LASSO was used to correct the risk of overfitting and narrowed the DEGs gene range, leaving eight genes to develop a prognostic model. The PRG score (risk score) was calculated by the following formula: Risk score = Σ (Expi * Coefi) (Coefi denotes the risk coefficient and Expi the gene expression). After risk calculation, we divided the patients into low-risk and high-risk groups according to the medium-risk value and then performed the survival analysis and receiver operating characteristic (ROC) curves in the training set. We also performed validation in the testing set and entire sets.

### Real-Time Quantitative PCR

Paired tumor and adjacent nontumor samples of prostate cancer tissues were obtained from the Beijing Hospital, Urology Department. The samples were preserved in the Biological Sample Center of Beijing Hospital and the Ethics Committee of the Beijing Hospital permitted the use of samples in this study.

The total RNA was extracted with the trizol (Invitrogen, America) reagent; the quantity and quality were evaluated *via* spectrophotometry. Reverse transcription was conducted using the reverse transcription system (A3500, Promega, America). We used SYBR Green Premix Pro Taq HS qPCR Kit (Accurate Biology) and MA-6000 real-time fluorescence quantitative PCR Detection System to perform the RT-qPCR process. The cycling conditions were 95°C for 10 min followed by 40 cycles of 95°C for 30 s, 60°C for 45 s, and 72°C for 45 min. The data analysis was conducted using the 2^-ΔΔ*C*^_t_ method. The sequence of the primers is listed in Supplementary Table S1.

### Relationship of the PRG Score With Prognosis Clinical Features, TME, CSC, and Mutation

In order to explore the clinical values of the two gene clusters, we compared patient characteristics including the tumor T stage, Gleason score, and prognosis. The Kaplan–Meier curve was generated with R packages “survival” and “survminer,” and the biochemical relapse (BRC) difference between the two clusters was determined. The ESTIMATE algorithm, a popular enrichment algorithm, which was extensively utilized in medical studies (Liu et al., 2021, 2022a, 2022b, 2022c), was used to evaluate the immune and stromal scores of each patient. We used the single-sample gene set enrichment analysis (ssGSEA) algorithm to evaluate the immune cell infiltration in the tumor microenvironment (TME). To assess the proportion of tumor immune infiltrating cells in tumor TME, the CIBERSORT algorithm was used in the low-risk and high-risk groups. We explored the association between the risk scores of 23 infiltrating immune cells and eight BCR-associated pyroptosis-related DEGs. In addition, we analyzed the relationship between the CSC and two risk groups. To determine the somatic mutations between the high-risk and low-risk groups, we applied the “maftools” R package to explore the mutation annotation format (MAF) from the TCGA database. We also calculated the tumor mutation burden (TMB) score in the two groups.

### Statistical Analysis

All statistical analyses were performed with software R (version 4.1.2), and Prism 8.0. Statistical significance was set at *p* < 0.05.

## Results

### The CNV and mRNA Alteration of PRGs in Prostate Cancer

**FIGURE 1:**
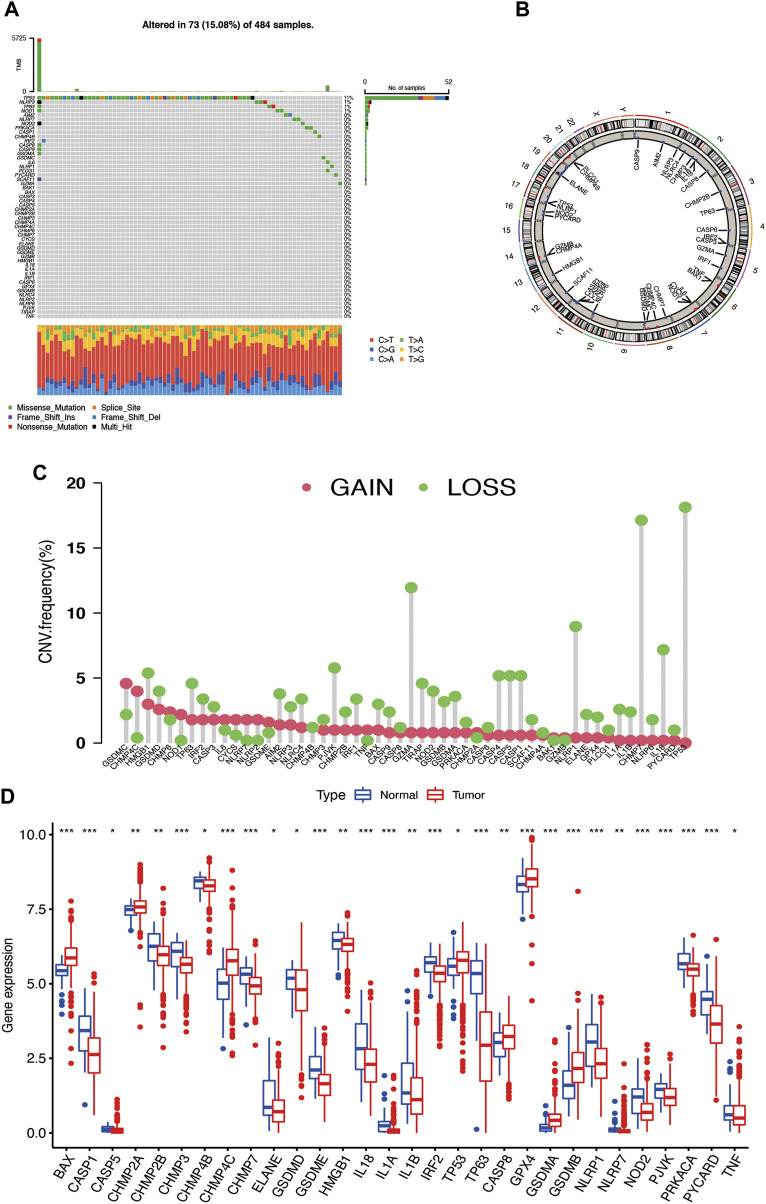
**(A)** Somatic mutation of PRGs in prostate cancer. **(B)** Copy number variation of PRGs in prostate cancer somatic cells. **(C)** The localization of CNV in chromosomes. **(D)** The differential expression of PRGs in tumor and normal tissues.

The flow chart of this research is shown in Supplementary Figure S1. The somatic mutation incidence of total 52 PRGs (Chen et al., 2021; Ye et al., 2021; Zhuang et al., 2021) in prostate cancer is shown in Figure 1A, and it was found that 73 prostate cancer samples (15.08%) of TCGA occurred with pyroptosis-related genes mutation, with TP53 having the highest mutation frequency (11%) followed by NLRP3 (1%), TP63 (1%), and NOD1 (1%). We also explored the copy number variation (CNV) of PRGs in prostate cancer and found universal copy number changes; the CNVs of GSDMD, GSDMC, HMGB1, CHMP4C, CHMP6, and NOD1 were increased, while those of TP53, IL18, NLRP1, HBGB1, GZMA, PJVK, CASP1, CASP5, CASP4, and CHMP7 were decreased (Figure 1B). The localization of CNV on chromosomes of each gene was also analyzed (Figure 1C). Then, we observed the relationship of PRG mRNA expression levels between tumor and normal tissues and found that the CNV-decreased PRGs, such as IL18, NLRP1, and PJVK, are highly expressed in the normal prostate tissue, while the CNV-increased PRGs, like CHMP4C, are upregulated in the tumor tissues (Figure 1D), suggesting that CNV may regulate the mRNA expression of PRGs. There are also a considerable number of PRGs like TP53, CHMP2A, GPX4, GSMDA, and GSMDB, that have opposite tendencies in mRNA expression and CNV trend. It is suggested that CNV may relate to PRG mRNA expression. It is known that the gene expression level is regulated by many factors, and the opposite trend of CNV and mRNA expression may involve other factors. The abovementioned results indicated that there is a significant difference in the genetic landscape and expression levels of PRGs in the tumor and normal samples, indicating that PRGs may play certain roles in prostate cancer progression.

### Identification of Pyroptosis Subtypes and Their TME Characteristics in Prostate Cancer

**FIGURE 2:**
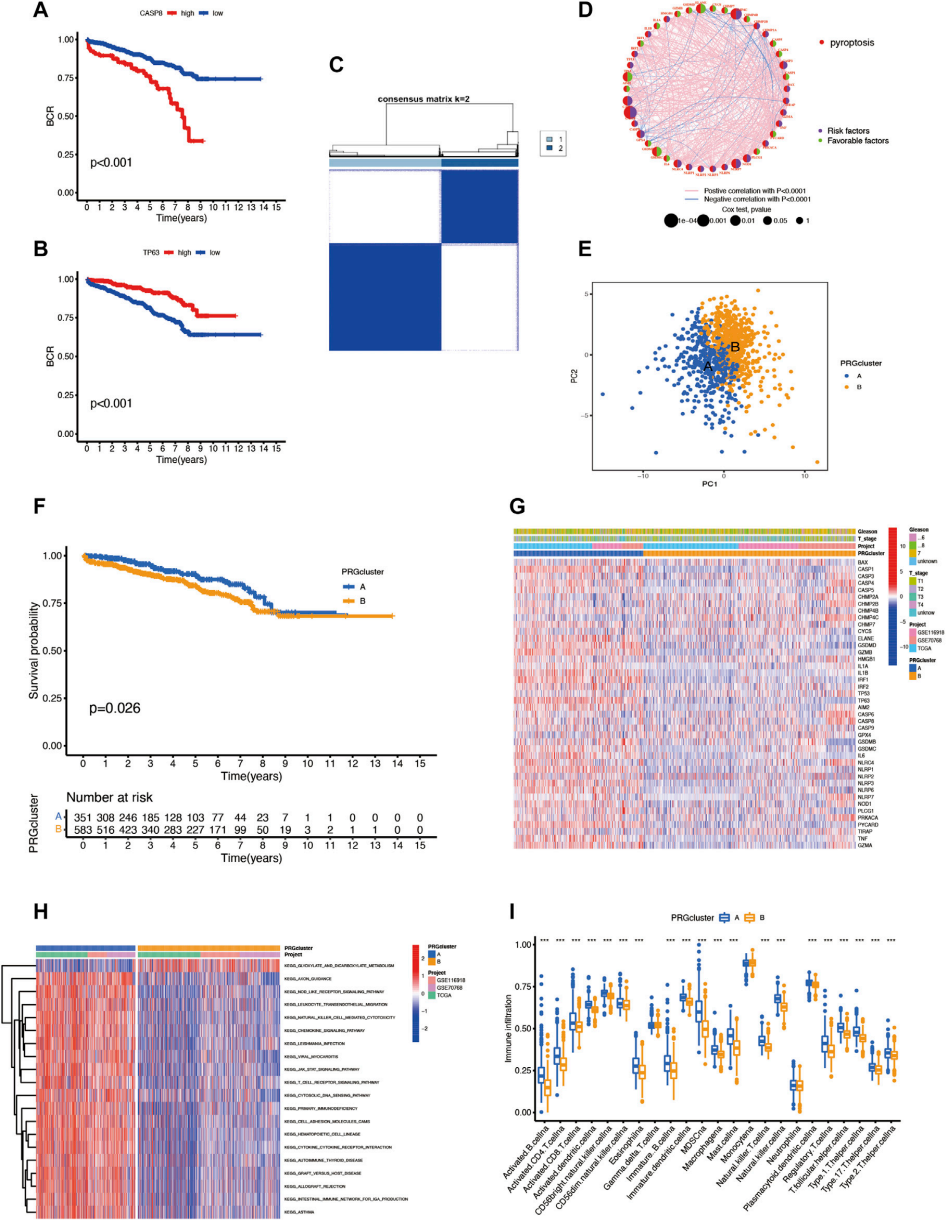
**(A,B)** The survival analysis of some PRGs. **(C)** Consensus clustering analysis classifies patients into two subtypes. **(D)** Pyroptosis network diagram. **(E)** PCA of two clusters. **(F)** Prognosis difference between subtypes A and B. **(G)** The heatmap reveals the relationship between clinical features and different subtypes. **(H)** GSVA enrichment analysis between two subtypes. **(I)** Immune cell infiltrates between the two subtypes.

We combined three different datasets (TCGA-PRAD, GSE70768, and GSE116918) into an entire cohort containing 1,026 patients. The Kaplan–Meier analysis was performed, and the results showed the prognosis of partial PRGs in prostate cancer (Figures 2A,B). Given the properties of prostate cancer, we chose biochemical recurrence as a prognosis indicator in this research; the pyroptosis network diagram exhibited interaction between PRGs and prognostic value in prostate cancer (Figure 2D). Next, we applied the consensus clustering analysis to classify the entire cohort into subtype A (*n* = 422) and subtype B (*n* = 604) based on the mRNA expression of 52 PRGs (Figure 2C) and PCA showing significant transcription profile differences between A and B subtypes (Figure 2E). The Kaplan–Meier curve showed that subtype A has a better survival probability than subtype B (*p* = 0.026; Figure 2F), and the two subtypes have no significant link with the clinical T stage and Gleason score (Figure 2G). In addition, the GSVA enrichment analysis revealed that subtypes A are enriched in many signalings like the axon guidance, nod-like receptor signaling pathway, leukocyte transendothelial migration, and natural killer cell-mediated cytotoxicity. (Figure 2H). To further understand the function of PRGs in TME, we used the CIBERSORT algorithm to evaluate the correlation between 23 human immune cell subpopulations and two subtypes and found significant differences in most immune cell infiltrates between the two subtypes. The immune cells, like activated B cells, activated CD4T cells, activated CD8T cells, and activated dendritic cells, were highly infiltrated in subtype A, but the gamma delta T cells, monocytes, and neutrophils were infiltered in subtype B (Figure 2I).

### Characteristics of Pyroptosis-Related DEGs

**FIGURE 3:**
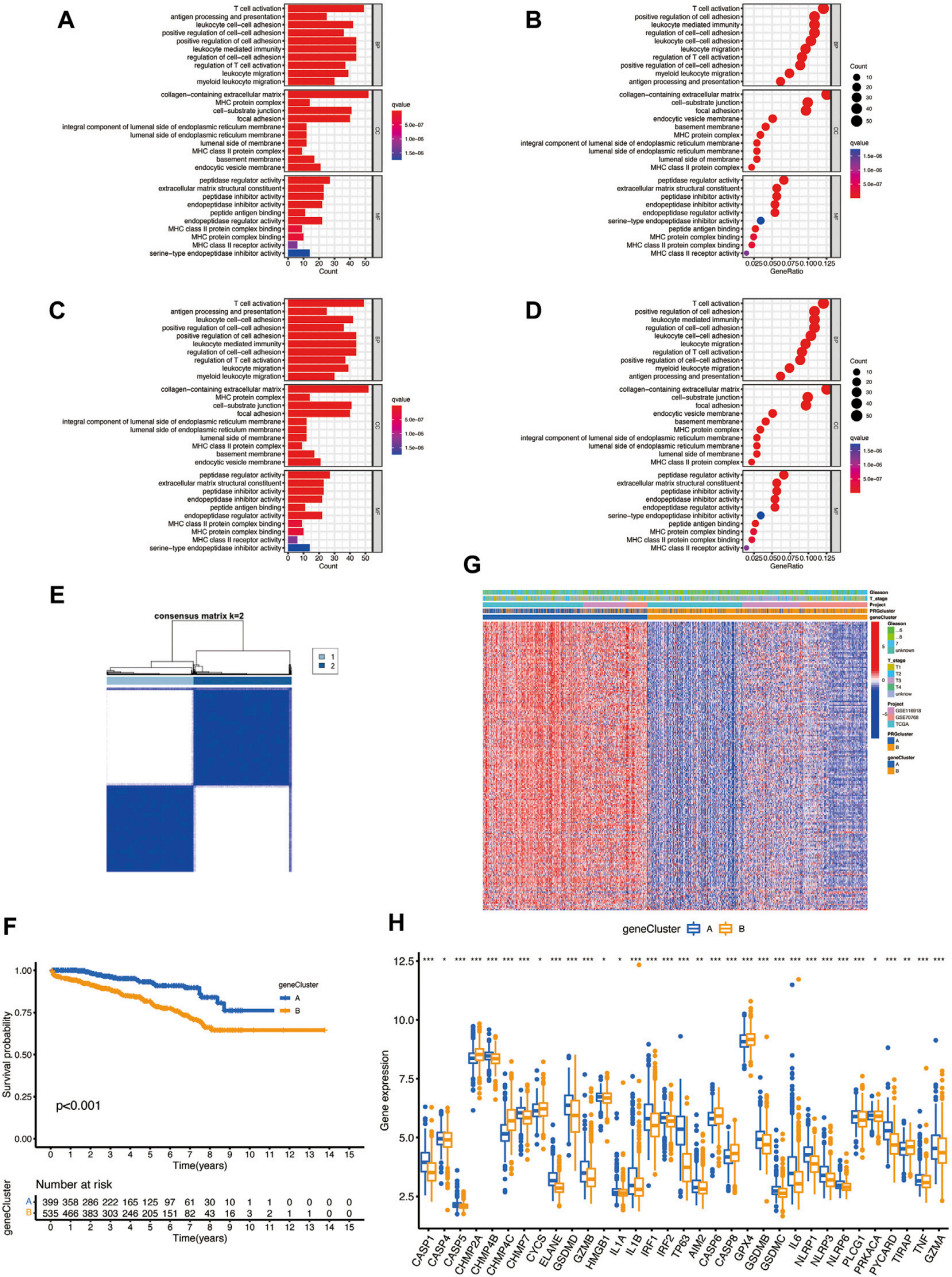
**(A–D)** The possible pathway associated with DEGs of two subtypes by KEGG and GO analysis. **(E)** Consensus clustering analysis divides patients into two gene clusters. **(F)** Prognosis of two clusters. **(G)** The heatmap shows the relationship between clinical features and two clusters. **(H)** Differential expression of PRGs between two clusters.

To explore the underlying biological behavior of pyroptosis in each subtype, we used the R package “limma” to identify 423 DEGs between the two subtypes and obtained functional enrichment analysis results (Figures 3A–D). The KEGG and GO analysis illustrated that DEGs were significantly enriched in the immune-related physiological processes, indicating that pyroptosis may play a vital role in prostate cancer immunomodulatory. We then performed univariate Cox regression in 423 BCR-related DEGs and obtained 217 DEGs (*p* < 0.05) for subsequent analysis (Supplementary Table S2). To further verify the regulatory mechanism, we used the consensus clustering algorithm to divide the patients into clusters A and B according to the prognostic results. The survival analysis shows that cluster B has a higher incidence of BCR events (*p* < 0.001; Figures 3E,F); however, the heatmap showed the that Gleason score and clinical T stage have no significant meaning in different clusters (Figure 3G). The two clusters showed significant differences in the expression of PRGs; CHMP2A, CHMP4C, CYCS, CASP6, CASP8, GPX4, and TIRAP have high expression levels in cluster B, suggesting that these genes may associate with poor prognosis in prostate cancer (Figure 3H).

### Establishment of the Pyroptosis Prognosis Model and Validation Based on Pyroptosis-Related DEGs

**FIGURE 4:**
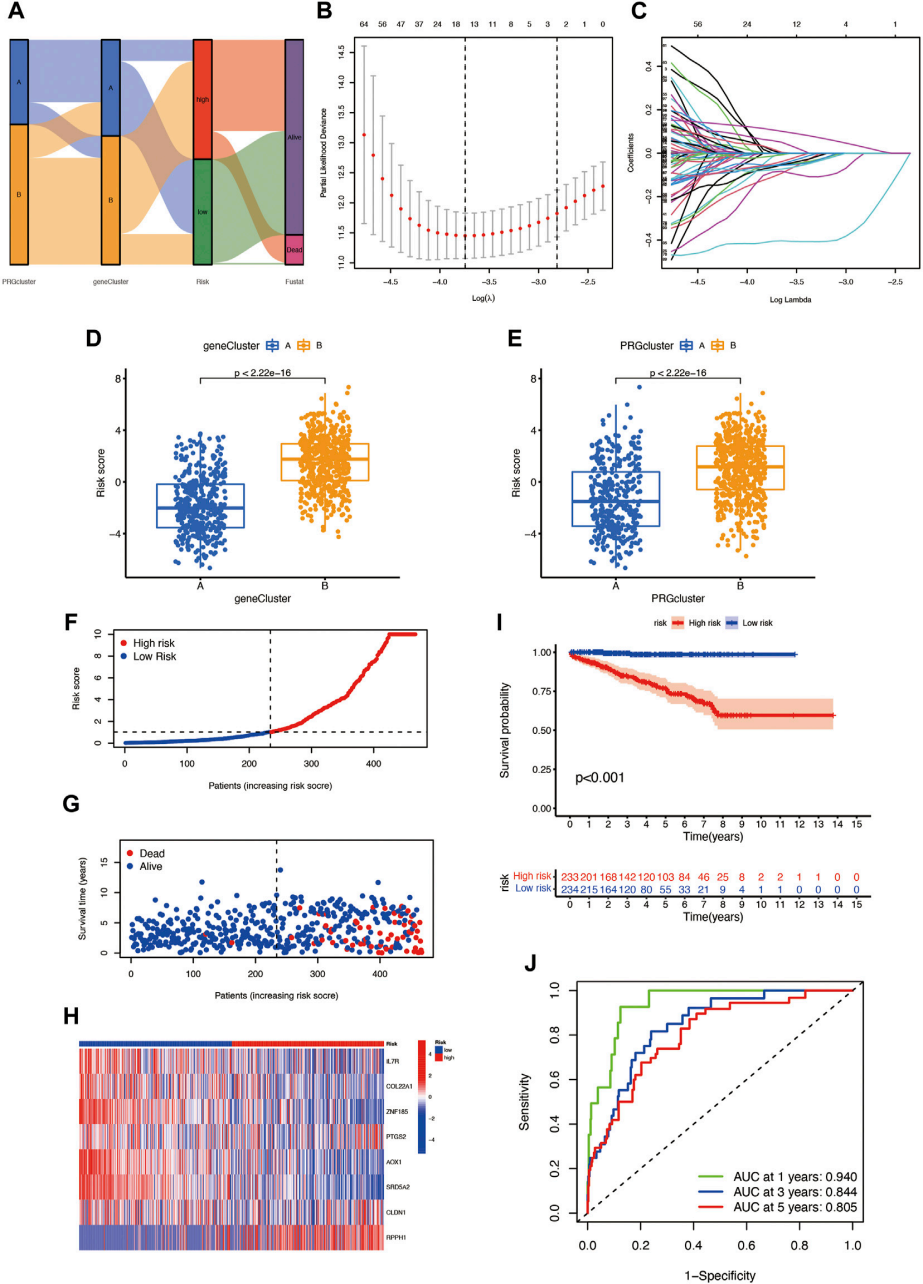
**(A)** Distribution of patients in different groups. **(B,C)** LASSO (least absolute shrinkage and selection operator) regression. **(D,E)** Different gene clusters and subtypes have significant differences in high- and low-risk score groups. **(F,G)** Risk score plots in the training set. **(H)** Risk heatmap of the two risk groups. **(I)** The BCR event occurrence rate of two risk groups in the training set. **(J)** The 1-, 3-, and 5-year BCR rates of different risk groups in the training group; AUC values were 0.940, 0.844, and 0.805, respectively.

The PRG score was established based on the pyroptosis-related DEGs. Figure 4A presents the distribution of two subtypes, two gene clusters, and two PRG score (risk score) groups. We randomly divided the entire cohort into the training (*n* = 467) and testing (*n* = 467) sets and performed LASSO analysis on 217 DEGs, previously obtained to further select the best prognostic signatures. After LASSO regression, 15 BCR-associated genes were selected (Figures 4B,C). Then, multivariate Cox regression analysis was performed among the 15 BCR-related genes and finally obtained eight genes (IL7R, COL22A1, ZNF185, PTGS2, AOX1, SRD5A2, CLDN1, and RPPH1), including three high-risk genes (PTGS2, CLDN1, and RPPH1) and five low-risk genes (IL7R, COL22A1, ZNF185, SRD5A2, and AOX1) (Supplementary Table S1). The risk score was calculated as follows: Risk score = (−0.4686 * IL7R expression) + (−0.3143 * COL22A1 expression) + (−0.6140 * ZNF185 expression) + (0.2191 * PTGS2 expression) + (−0.3185 * AOX1 expression) + (−0.6888 * SRD5A2 expression) + (0.2849 * CLDN1 expression) + (0.2189 * RPPH1 expression). Obvious risk score differences were observed in the two clusters: cluster A had a lower risk score while cluster B had a higher risk score (Figure 4D), and the distribution of risk scores for the two subtypes is shown in Figure 4E. We divided the patients into high- and low-risk groups according to the median risk score in both the training sets and found that the BCR incidence positively correlated to the risk score (Figures 4F,G). The risk heatmap showed that RPPH1, CLDN1, and PTGS2 are upregulated as the risk score increases (Figure 4H). The survival analysis revealed a significant difference between the high- and low-risk groups in the training set (Figure 4I, *p* < 0.001). The 1-, 3-, and 5-year BCR rates of the different risk scores in the training set are represented by the AUC values in Figure 4J. The survival analysis showed that the prognosis of the low-risk was significantly better than that of the high-risk group, indicating that the PRG score had an excellent ability to predict the BCR event occurrence rate in prostate cancer.

### Validation in Real Samples

**FIGURE 5:**
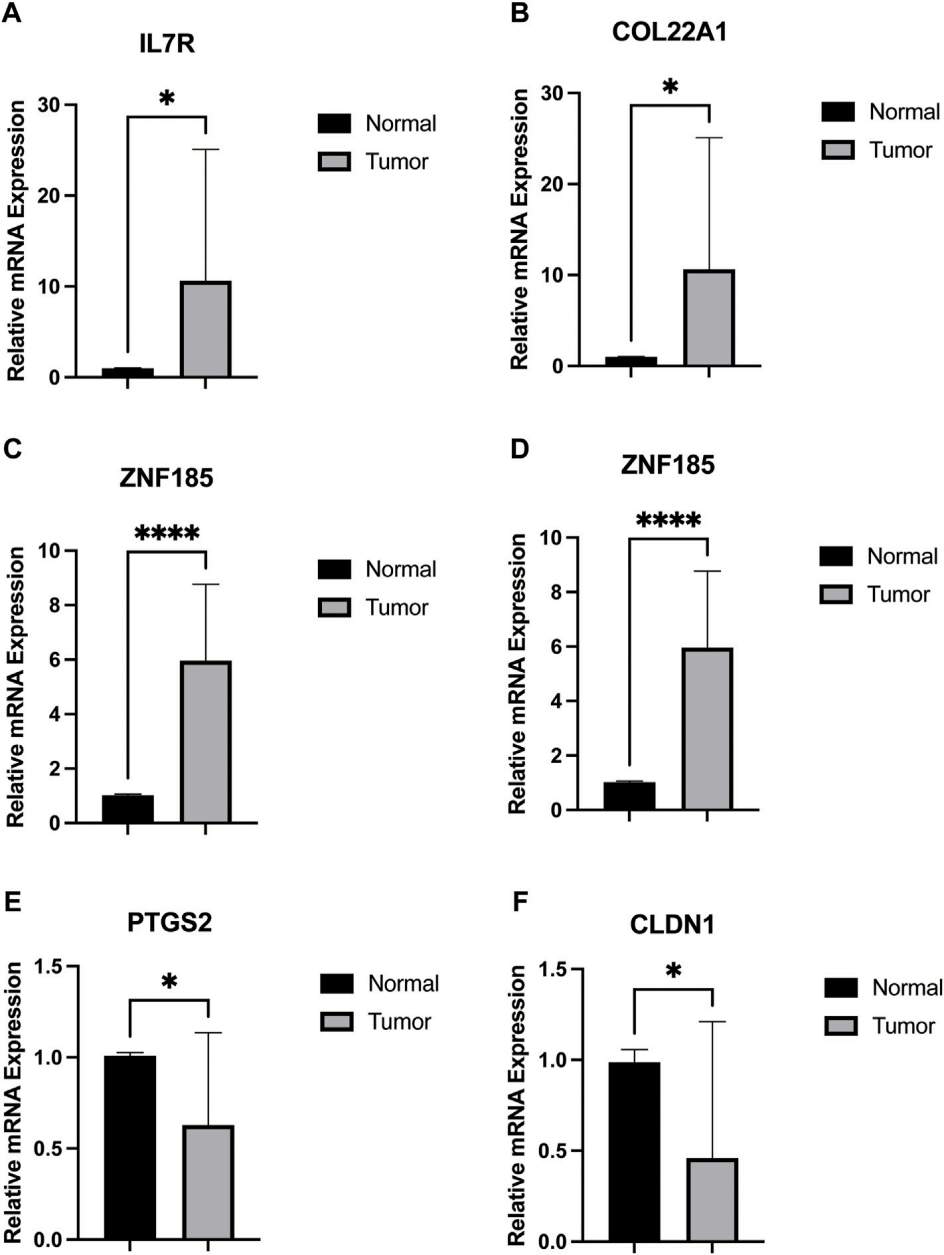
mRNA expression of 12 paired prostate tumors and normal tissues obtained from patients.

We obtained 12-paired tumor and normal prostate cancer tissues from the Beijing Hospital, Urology Department, and performed the RT-qPCR. We found that the mRNA expression of IL7R, COL22A1, ZNF185, and SRD5A2 were upregulated in cancer tissues, while PTGS2 and CLDN1 were higher in normal tissues (Figure 5), consistent with our previous analysis.

### Validation of the Prognostic Model in the Entire and Testing Sets

**FIGURE 6:**
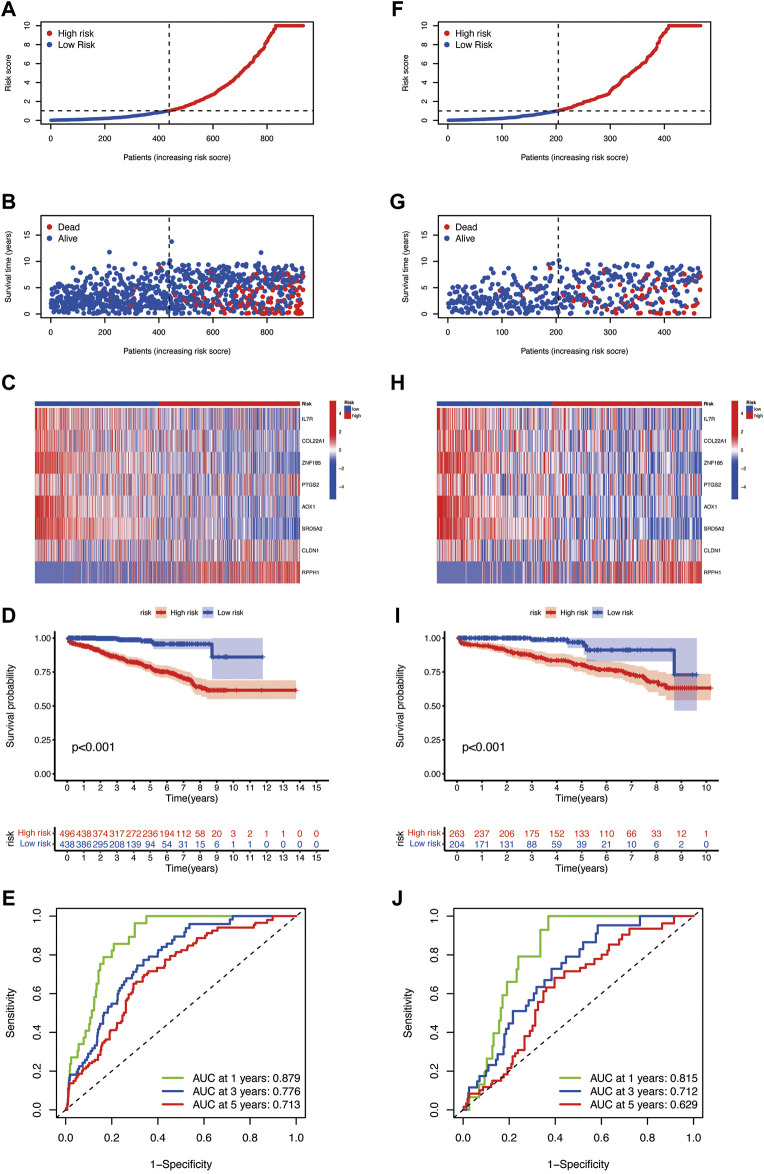
Risk plots, the risk heatmap, the BCR incidence plot, and the 1-, 3-, and 5-year BCR rates of different risk score groups in the **(A–E)** entire cohort and **(F–J)** the testing set.

To verify the prognostic prediction value of the risk model, we performed the same steps in the entire set (Figures 6A–D)and testing set (Figures 6F–I) and obtained the same meaningful results as in the training set, the risk score plots, and survival plots. The ROC curves of the entire cohort (Figure 6E) and the testing set (Figure 6J) were performed to predict the sensitivity and specificity of 1-, 3-, and 5-year BCR rates of different risk scores; the AUC values of 1-, 3-, and 5-year BCR occurrence in the entire cohort are 0.897, 0.776, and 0.713, respectively, and 0.815, 0.712, and 0.629 in the testing set, respectively.

### Evaluation of TME in Different Risk Groups

**FIGURE 7:**
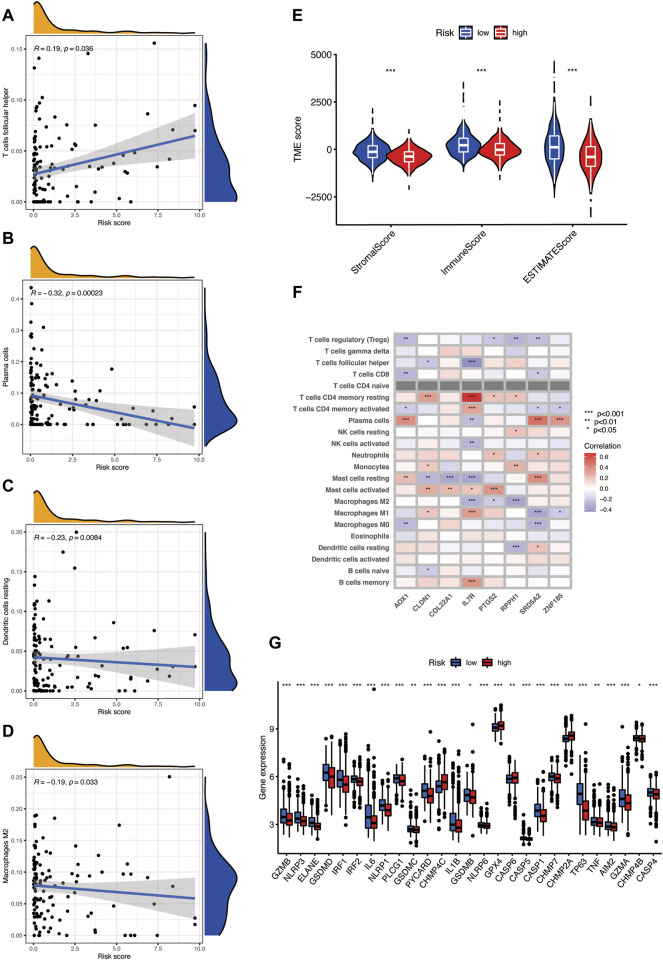
**(A–D)** The risk score has a positive correlation with the T-cell follicular helper and a negative correlation with dendritic cell resting, macrophages M2, and plasma cells, as found using the CIBERSORT algorithm. **(E)** TME scores in the two risks groups, including the stromal score, immune score, and ESTIMATE score. **(F)** The relationship between eight selected PRGs and the abundance of immune cells. **(G)** The expression level of pyroptosis-related genes in two risk groups.

We used the CIBERSORT algorithm to evaluate the relationship between the risk score and immune cell abundance. Figures 7A–D show that the risk score has a positive correlation with the T-cell follicular helper and a negative correlation with the dendritic cell resting, macrophages M2, and plasma cells. In addition, we assess the TME scores including the stromal score, immune score, and ESTIMATE score. The violin plot shows that the scores of the low-risk group are significantly higher among the three TME scores, higher stromal score, or immune score, indicating an abundant relative concentration of stromal or immune cells in the prostate tumor microenvironment, and the ESTIMATE score suggests the aggregation of stromal or immune scores in the TME (Figure 7E). We also evaluated the relationship between the finally selected eight genes in the model and the abundance of immune cells and found that the eight genes are significantly correlated with different immune cells (Figure 7F). We then investigated the association between PRGs and our risk model. Figure 7G shows the PRG expression in two risk groups, and most genes have significant expression differences; the expression of CHMP4C, GPX4, CASP6, and CHMP2A was increased in the high-risk group, and the others expressed higher in the low-risk group.

### The Relationship of Risk Score With the CSC Index and Mutation

**FIGURE 8:**
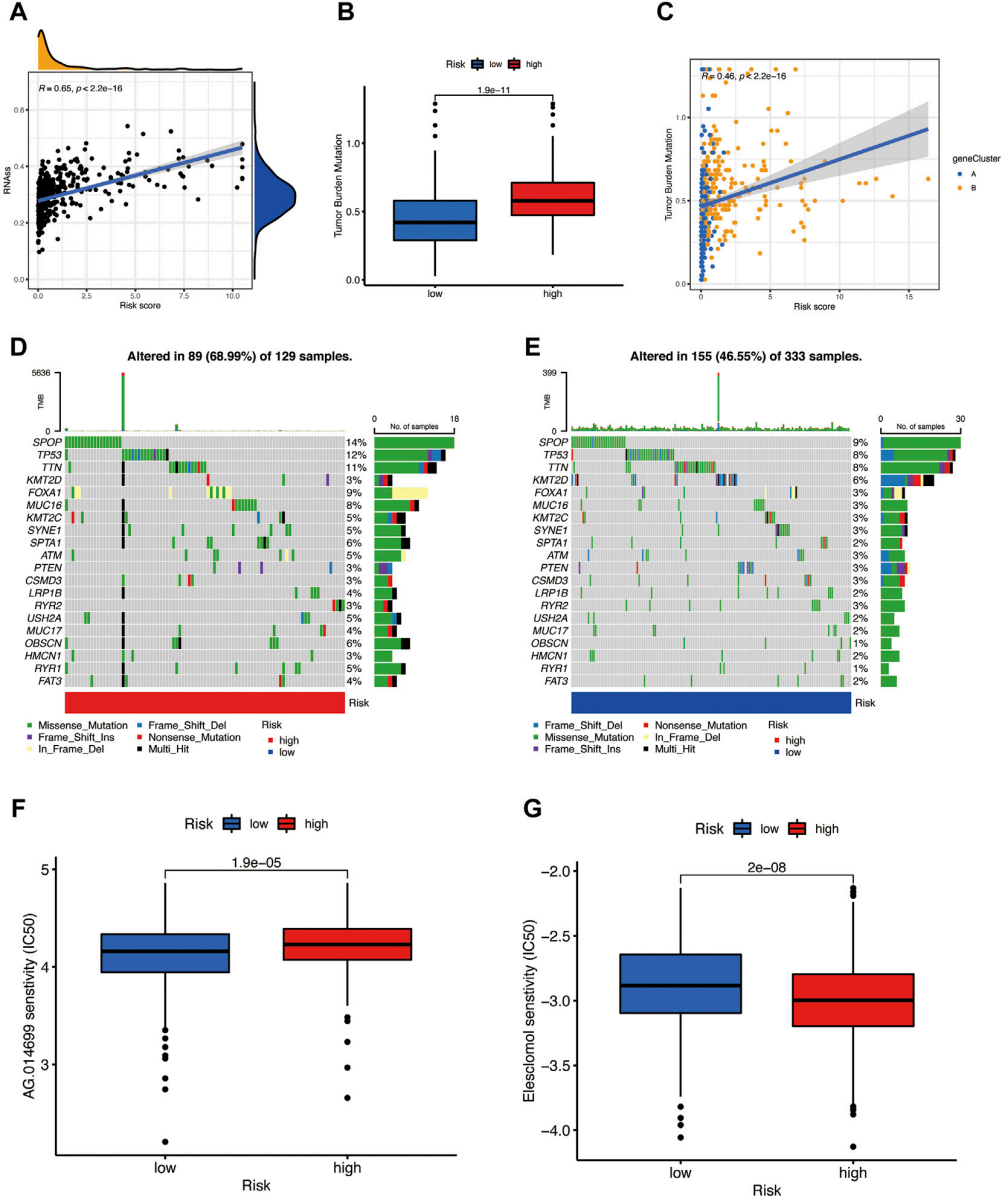
**(A)** The risk score has a positive relationship with the CSC index. **(B,C)** Boxplot and Spearman correlation reveal that the high-risk group has a higher TME rate. **(D)** Somatic mutation of pyroptosis-related genes in a high-risk group. **(E)** Somatic mutation of PRGs in the low-risk group. **(F)** Rucaparib is sensitive in different risk groups. **(G)** Elesclomol is sensitive in different risk groups.

The risk score has a positive linear correlation with the CSC index (Figure 8A, R = 0.65, *p* < 0.001), indicating that prostate cancer cells with a high-risk score have higher stem cell characteristics. According to previous studies, patients with high TMB may benefit from immunotherapy, and our analysis of TCGA-PRAD mutation found that the high-risk group has a higher TMB (Figure 8B, *p* < 0.001) and the Spearman correlation illustrated that the PRG risk score was positively related to TMB (Figure 8C, *R* = 0.46, *p* < 0.001), which means that the high-risk group may benefit from immunotherapy. Then, we analyzed the distribution of the somatic mutations in two TCGA-PRAD cohort risk groups. The top ten mutant genes in both the high-and the low-risk groups are SPOP, TP53, TTN, KMT2D, FOXA1, MUC16, KMT2C, SYNE1, SPTA1, ATM, and PTEN (Figures 8D,E). Compared with the low-risk scores, the high-risk patients had higher mutation frequencies of SPOP, TP53, and TTN, and lower mutation of KMT2D; the mutation frequencies of PTEN, RYR2, and CSMD3B were almost similar between the two risk groups (Figures 8D,E). In addition, we observed that drugs like Rucaparib (Figure 8F) have a significantly higher IC50 in the high-risk score; in contrast, the IC50 of Elesclomol (Figure 8G) evidently decreased in the high-risk score group. This may suggest that PRGs have a close relationship with drug sensitivity and may guide the chemotherapeutic or immunotherapy in high TMB patients.

## Discussion

Pyroptosis is an emerging concept, and the caspases cleave gasdermin D (GSDMD is a member belonging to gasdermin family (Shi et al., 2015; Wang et al., 2017)). Pyroptosis plays an indispensable role in inflammation, tumor immunity modulation, and tumor progression. However, there are few discussions about PRGs in prostate cancer. There are many causes of prostate cancer, and inflammation plays a certain role in the occurrence and progression of prostate cancer but the mechanism is not yet clear. As a kind of programmed cell death, pyroptosis causes excessive inflammation of cells when the cell receives a stimulus, which subsequently causes the recruitment of immune cells and results in cell death. Previous studies have shown that pyroptosis plays different regulation roles in tumor progression, either inhibiting or promoting, but the role of pyroptosis in the progression of prostate cancer is not yet known. Therefore, this article focuses on exploring the role of PRGs in prostate cancer biochemical recurrence. We construct a risk model to predict the biochemical relapse events in prostate cancer. The results show many CNV and mRNA alterations of PRGs in prostate cancer. According to the expression level of PRGs in prostate cancer, we have identified subtypes A and B and explored the difference between the two subtypes. We found that in the TCGA and GEO databases, PRGs have significant prognostic predictive ability, and the expressions of CASP8, CHMP4C, HMGB1, GSDMB, GSDMC, ELANE, NLRP7, and other genes in prostate cancer have a significant correlation with the BCR, Also, subtype A has a better BCR-free survival rate, and it was found that the two subtypes have significant immune activation and related immune pathways are activated. We then obtained the DEGs between subtypes A and B. After the Lasso and multivariate cox regression analysis of the DEGs, eight signatures were selected and the risk scores model was constructed. Based on the median risk score, all prostate cancer patients were divided into high- and low-risk groups. The patients in the low- and high-risk groups represented significantly different prognosis, mutations, TME and CSC indexes, and drug sensitivity. Therefore, we envisage that the risk model can guide clinical decision-making in a targeted manner in prostate cancer patients.

The TME is closely related to tumor progression (Hinshaw and Shevde, 2019) and also plays an important role in the metastasis and progression of prostate cancer (Zeng et al., 2006). In our study, according to KEGG and GO analysis, the DEGs of subtypes A and B are enriched in the immune-related pathway, and participated in the immune reaction of TME, like the processes of inflammation, cell adhesion, and leukocyte migration. Then we constructed the risk score model and divided all data into the high- and low-risk group. After that, we use the ESTIMATE algorithm to calculate the stromal score, immune score, and ESTIMATE (immune/stromal) score, and the results exhibit the poor prognosis related to lower TME scores, that is a higher BCR event rate. The predictive model established by eight PRG signatures represented the low-risk group having a better prognosis, and the 1-,3-, and 5-year AUC values of the training set were 0.940, 0.844, and 0.805, respectively, and the 1-,3-, and 5-year AUC values of the test set and entire cohort also had ideal results. Therefore, this model is expected to be applied to the assessment of biochemical recurrence of prostate cancer patients.

In our risk model, we finally selected eight PRG signatures, of which IL7R, COL22A1, ZNF185, SRD5A2, and AOX1 have a positive correlation with the prognosis, while PTGS2, CLDN1, and RPPH1 have a negative correlation with the prognosis. Pyroptosis plays an important role in both promoting and inhibiting cancer progression. On the one hand, the release of a large number of inflammatory factors caused by pyroptosis will promote the transformation of normal cells into tumor cells (Karki and Kanneganti, 2019); on the other hand, pyroptosis can induce the death of tumor cells (Xia et al., 2019). Il7R and COL22A1 are related to the TME of lung cancer (Fan et al., 2021) and breast cancer (Ademuyiwa et al., 2021), respectively. Zinc finger protein 185 (ZNF185) is a novel LIM domain gene and is rarely expressed in most human tissues of the body; however, it is most abundantly expressed in the prostate (Heiss et al., 1997; Zhang et al., 2007). The expression level of ZNF15 has a negative correlation with prostate cancer progression, and the methylation of the 5′CpG islands of the ZNF185 gene has a close relationship with metastasis of prostate cancer (Vanaja et al., 2003). ZNF185 is verified in databases like TCGA and GEO that are upregulated in the normal prostate epithelial cells (Smirnov et al., 2018), ZNF185 mutation is associated with metastasis behavior in breast cancer (Krøigård et al., 2018). Human steroid 5α-reductase 2 (SRD5A2) is related to the progression of prostate cancer and benign prostatic hyperplasia (Xiao et al., 2020). It can catalyze the conversion of testosterone to dihydrotestosterone, and finasteride can inhibit the expression of ARD5A2 (Azzouni et al., 2012; Robitaille and Langlois, 2020). A recent study has found that the methylation of SRD5A2 is related to the biochemical recurrence of prostate cancer, which may be applied to predict the BCR (Horning et al., 2015). However, the SNP rs73055188 at the AOX1 locus is related to the survival time of prostate cancer, and lower expression of AOX1 is associated with early biochemical recurrence events (Li et al., 2018). Also, the AOX1 CpG island methylation is highly detected in prostate cancer and can be used as a prognostic signature (Haldrup et al., 2013; Shui et al., 2016). According to the previous literature, the positive prognostic values of ZNF185, SRD5A2, and AOX1 are expressed in normal tissues than in tumors, but their methylation levels in tumor tissues are higher than those in normal tissues. Therefore, methylation of PRGs may be a risk factor for the prostate cancer prognosis. On the contrary, PTGS2, CLDN1, and RPPH1 are negatively correlated with the prognosis of prostate cancer.

PTGS2 is the gene encoding COX2, and methylation of it is an independent prognostic indicator of biochemical recurrence in patients with local prostate cancer (Woodson et al., 2006). The CpG island hypermethylation heralds an increased risk of prostate cancer recurrence (Yegnasubramanian et al., 2004; Bastian et al., 2005; Phé et al., 2010; Moritz et al., 2013), and inhibiting PTGS2 in pancreatic cancer will reverse T-cell exclusion and sensitized tumors to immunotherapy (Markosyan et al., 2019), indicating that PTGS2 has a relationship with the tumor microenvironment (TME) in certain cancer. CLDN1, as a downstream gene of NF-κB, promotes epithelial–mesenchymal transition of lung adenocarcinoma (Guo et al., 2017). The high expression level of CLDN1 in ovarian cancer is related to tumor invasion and recurrence, and CLDN1 methylation can inhibit its expression level (Visco et al., 2021), which means that CLDN1 may be a poor prognosis factor in many cancer types, and methylation is a protective ingredient, which is completely opposite to the five genes that we have screened associated to a positive prognosis. RPPH1 is a long noncoding RNA and facilitates cancer development of many cancers like esophageal cancer (Li et al., 2020) and breast cancer (Zhang and Tang, 2017) and also receives mediated macrophages to promote the proliferation and metastasis of colorectal cancer (Liang et al., 2019).

All the data in this study are obtained from public databases and without permission to obtain certain specific clinical data, so there are some limitations in our studies. If we could validate this conclusion from multiple clinical data or conduct some prospective studies, simultaneously including more clinical features related to prostate cancer, such as the relationship between different drug treatments and the expression level of PRGs, this article will be more substantial.

## Conclusion

Our study established and validated a risk model based on eight pyroptosis-related genes, provided some evidence for predicting the BCR of prostate cancer, and further proved that pyroptosis is related to tumor immune cell infiltration in prostate cancer. These genes may be important in guiding the application of prostate cancer immunotherapy and the prediction of BCR events.

## Data Availability

The original contributions presented in the study are included in the article/Supplementary Material. Further inquiries can be directed to the corresponding author.

## References

[B1] Ademuyiwa F. O. Chen I. Luo J. Rimawi M. F. Hagemann I. S. Fisk B. (2021). Immunogenomic Profiling and Pathological Response Results from a Clinical Trial of Docetaxel and Carboplatin in Triple-Negative Breast Cancer. Breast Cancer Res. Treat. 189 (1), 187–202. 10.1007/s10549-021-06307-3 34173924 PMC8443324

[B2] Azzouni F. Godoy A. Li Y. Mohler J. (2012). The 5 Alpha-Reductase Isozyme Family: a Review of Basic Biology and Their Role in Human Diseases. Adv. Urol. 2012, 530121. 10.1155/2012/530121 22235201 PMC3253436

[B3] Bastian P. J. Ellinger J. Wellmann A. Wernert N. Heukamp L. C. Müller S. C. (2005). Diagnostic and Prognostic Information in Prostate Cancer with the Help of a Small Set of Hypermethylated Gene Loci. Clin. Cancer Res. 11 (11), 4097–4106. 10.1158/1078-0432.ccr-04-1832 15930345

[B4] Bray F. Ferlay J. Soerjomataram I. Siegel R. L. Torre L. A. Jemal A. (2018). Global Cancer Statistics 2018: GLOBOCAN Estimates of Incidence and Mortality Worldwide for 36 Cancers in 185 Countries. CA A Cancer J. Clin. 68 (6), 394–424. 10.3322/caac.21492 30207593

[B5] Chen W. Zhang W. Zhou T. Cai J. Yu Z. Wu Z. (2021). A Newly Defined Pyroptosis-Related Gene Signature for the Prognosis of Bladder Cancer. Int. J. Gen. Med. 14, 8109–8120. 10.2147/ijgm.s337735 34803395 PMC8594790

[B6] Cookson B. T. Brennan M. A. (2001). Pro-inflammatory Programmed Cell Death. Trends Microbiol. 9 (3), 113–114. 10.1016/s0966-842x(00)01936-3 11303500

[B7] Fan T. Pan S. Yang S. Hao B. Zhang L. Li D. (2021). Clinical Significance and Immunologic Landscape of a Five-IL(R)-Based Signature in Lung Adenocarcinoma. Front. Immunol. 12, 693062. 10.3389/fimmu.2021.693062 34497605 PMC8419226

[B8] Fang Y. Tian S. Pan Y. Li W. Wang Q. Tang Y. (2020). Pyroptosis: A New Frontier in Cancer. Biomed. Pharmacother. 121, 109595. 10.1016/j.biopha.2019.109595 31710896

[B9] Guo J. Y. Hsu H. S. Tyan S. W. Li F. Y. Shew J. Y. Lee W. H. (2017). Serglycin in Tumor Microenvironment Promotes Non-small Cell Lung Cancer Aggressiveness in a CD44-dependent Manner. Oncogene 36 (17), 2457–2471. 10.1038/onc.2016.404 27819672 PMC5415946

[B10] Ha Chung B. Horie S. Chiong E. (2019). The Incidence, Mortality, and Risk Factors of Prostate Cancer in Asian Men. Prostate Int. 7 (1), 1–8. 10.1016/j.prnil.2018.11.001 30937291 PMC6424686

[B11] Haldrup C. Mundbjerg K. Vestergaard E. M. Lamy P. Wild P. Schulz W. A. (2013). DNA Methylation Signatures for Prediction of Biochemical Recurrence after Radical Prostatectomy of Clinically Localized Prostate Cancer. J. Clin. Oncol. 31 (26), 3250–3258. 10.1200/jco.2012.47.1847 23918943

[B12] Heiss N. S. Gloeckner G. Bächner D. Kioschis P. Klauck S. M. Hinzmann B. (1997). Genomic Structure of a Novel LIM Domain Gene (ZNF185) in Xq28 and Comparisons with the Orthologous Murine Transcript. Genomics 43 (3), 329–338. 10.1006/geno.1997.4810 9268636

[B13] Hinshaw D. C. Shevde L. A. (2019). The Tumor Microenvironment Innately Modulates Cancer Progression. Cancer Res. 79 (18), 4557–4566. 10.1158/0008-5472.can-18-3962 31350295 PMC6744958

[B14] Horning A. M. Awe J. A. Wang C. M. Liu J. Lai Z. Wang V. Y. (2015). DNA Methylation Screening of Primary Prostate Tumors Identifies SRD5A2 and CYP11A1 as Candidate Markers for Assessing Risk of Biochemical Recurrence. Prostate 75 (15), 1790–1801. 10.1002/pros.23052 26332453

[B15] Jorgensen I. Rayamajhi M. Miao E. A. (2017). Programmed Cell Death as a Defence against Infection. Nat. Rev. Immunol. 17 (3), 151–164. 10.1038/nri.2016.147 28138137 PMC5328506

[B16] Karki R. Kanneganti T. D. (2019). Diverging Inflammasome Signals in Tumorigenesis and Potential Targeting. Nat. Rev. Cancer 19 (4), 197–214. 10.1038/s41568-019-0123-y 30842595 PMC6953422

[B17] Kovacs S. B. Miao E. A. (2017). Gasdermins: Effectors of Pyroptosis. Trends Cell Biol. 27 (9), 673–684. 10.1016/j.tcb.2017.05.005 28619472 PMC5565696

[B18] Krøigård A. B. Larsen M. J. Lænkholm A. V. Knoop A. S. Jensen J. D. Bak M. (2018). Identification of Metastasis Driver Genes by Massive Parallel Sequencing of Successive Steps of Breast Cancer Progression. PLoS One 13 (1), e0189887. 10.1371/journal.pone.0189887 29293529 PMC5749725

[B19] Li W. Middha M. Bicak M. Sjoberg D. D. Vertosick E. Dahlin A. (2018). Genome-wide Scan Identifies Role for AOX1 in Prostate Cancer Survival. Eur. Urol. 74 (6), 710–719. 10.1016/j.eururo.2018.06.021 30289108 PMC6287611

[B20] Li Z. Y. Li H. F. Zhang Y. Y. Zhang X. L. Wang B. Liu J. T. (2020). Value of Long Non-coding RNA Rpph1 in Esophageal Cancer and its Effect on Cancer Cell Sensitivity to Radiotherapy. World J. Gastroenterol. 26 (15), 1775–1791. 10.3748/wjg.v26.i15.1775 32351293 PMC7183868

[B21] Liang Z. X. Liu H. S. Wang F. W. Xiong L. Zhou C. Hu T. (2019). LncRNA RPPH1 Promotes Colorectal Cancer Metastasis by Interacting with TUBB3 and by Promoting Exosomes-Mediated Macrophage M2 Polarization. Cell Death Dis. 10 (11), 829. 10.1038/s41419-019-2077-0 31685807 PMC6828701

[B22] Liu Z. Guo C. Dang Q. Wang L. Liu L. Weng S. (2022). Integrative Analysis from Multi-Center Studies Identities a Consensus Machine Learning-Derived lncRNA Signature for Stage II/III Colorectal Cancer. EBioMedicine 75, 103750. 10.1016/j.ebiom.2021.103750 34922323 PMC8686027

[B23] Liu Z. Guo C. Li J. Xu H. Lu T. Wang L. (2021). Somatic Mutations in Homologous Recombination Pathway Predict Favourable Prognosis after Immunotherapy across Multiple Cancer Types. Clin. Transl. Med. 11 (12), e619. 10.1002/ctm2.619 34923755 PMC8684773

[B24] Liu Z. Liu L. Weng S. Guo C. Dang Q. Xu H. (2022). Machine Learning-Based Integration Develops an Immune-Derived lncRNA Signature for Improving Outcomes in Colorectal Cancer. Nat. Commun. 13 (1), 816. 10.1038/s41467-022-28421-6 35145098 PMC8831564

[B25] Liu Z. Xu H. Weng S. Ren Y. Han X. (2022). Stemness Refines the Classification of Colorectal Cancer with Stratified Prognosis, Multi-Omics Landscape, Potential Mechanisms, and Treatment Options. Front. Immunol. 13, 828330. 10.3389/fimmu.2022.828330 35154148 PMC8828967

[B26] Markosyan N. Li J. Sun Y. H. Richman L. P. Lin J. H. Yan F. (2019). Tumor Cell-Intrinsic EPHA2 Suppresses Anti-tumor Immunity by Regulating PTGS2 (COX-2). J. Clin. Invest. 129 (9), 3594–3609. 10.1172/jci127755 31162144 PMC6715369

[B27] Moritz R. Ellinger J. Nuhn P. Haese A. Müller S. C. Graefen M. (2013). DNA Hypermethylation as a Predictor of PSA Recurrence in Patients with Low- and Intermediate-Grade Prostate Cancer. Anticancer Res. 33 (12), 5249–5254. 24324057

[B28] Nakai Y. Nonomura N. (2013). Inflammation and Prostate Carcinogenesis. Int. J. Urol. 20 (2), 150–160. 10.1111/j.1442-2042.2012.03101.x 22852773

[B29] Phé V. Cussenot O. Rouprêt M. (2010). Methylated Genes as Potential Biomarkers in Prostate Cancer. BJU Int. 105 (10), 1364–1370. 10.1111/j.1464-410x.2009.09167.x 20067451

[B30] Robitaille J. Langlois V. S. (2020). Consequences of Steroid-5α-Reductase Deficiency and Inhibition in Vertebrates. Gen. Comp. Endocrinol. 290, 113400. 10.1016/j.ygcen.2020.113400 31981690

[B31] Shi J. Zhao Y. Wang K. Shi X. Wang Y. Huang H. (2015). Cleavage of GSDMD by Inflammatory Caspases Determines Pyroptotic Cell Death. Nature 526 (7575), 660–665. 10.1038/nature15514 26375003

[B32] Shui I. M. Wong C. J. Zhao S. Kolb S. Ebot E. M. Geybels M. S. (2016). Prostate Tumor DNA Methylation Is Associated with Cigarette Smoking and Adverse Prostate Cancer Outcomes. Cancer 122 (14), 2168–2177. 10.1002/cncr.30045 27142338 PMC4930391

[B33] Smirnov A. Cappello A. Lena A. M. Anemona L. Mauriello A. Di Daniele N. (2018). ZNF185 Is a P53 Target Gene Following DNA Damage. Aging (Albany NY) 10 (11), 3308–3326. 10.18632/aging.101639 30446632 PMC6286825

[B34] Van den Broeck T. van den Bergh R. C. N. Arfi N. Gross T. Moris L. Briers E. (2019), Prognostic Value of Biochemical Recurrence Following Treatment with Curative Intent for Prostate Cancer: A Systematic Review. Eur. Urol. 75(6):967–987. 10.1016/j.eururo.2018.10.011 30342843

[B35] Vanaja D. K. Cheville J. C. Iturria S. J. Young C. Y. (2003). Transcriptional Silencing of Zinc Finger Protein 185 Identified by Expression Profiling Is Associated with Prostate Cancer Progression. Cancer Res. 63 (14), 3877–3882. 12873976

[B36] Visco Z. R. Sfakianos G. Grenier C. Boudreau M. H. Simpson S. Rodriguez I. (2021). Epigenetic Regulation of Claudin-1 in the Development of Ovarian Cancer Recurrence and Drug Resistance. Front. Oncol. 11, 620873. 10.3389/fonc.2021.620873 33828978 PMC8019902

[B37] Wang Y. Gao W. Shi X. Ding J. Liu W. He H. (2017). Chemotherapy Drugs Induce Pyroptosis through Caspase-3 Cleavage of a Gasdermin. Nature 547 (7661), 99–103. 10.1038/nature22393 28459430

[B38] Woodson K. O'Reilly K. J. Ward D. E. Walter J. Hanson J. Walk E. L. (2006). CD44 and PTGS2 Methylation Are Independent Prognostic Markers for Biochemical Recurrence Among Prostate Cancer Patients with Clinically Localized Disease. Epigenetics 1 (4), 183–186. 10.4161/epi.1.4.3530 17998819

[B39] Xia X. Wang X. Cheng Z. Qin W. Lei L. Jiang J. (2019). The Role of Pyroptosis in Cancer: Pro-cancer or Pro-"host. Cell Death Dis. 10 (9), 650. 10.1038/s41419-019-1883-8 31501419 PMC6733901

[B40] Xiao Q. Wang L. Supekar S. Shen T. Liu H. Ye F. (2020). Structure of Human Steroid 5α-Reductase 2 with the Anti-androgen Drug Finasteride. Nat. Commun. 11 (1), 5430. 10.1038/s41467-020-19249-z 33110062 PMC7591894

[B41] Xu Y. J. Zheng L. Hu Y. W. Wang Q. (2018). Pyroptosis and its Relationship to Atherosclerosis. Clin. Chim. Acta 476, 28–37. 10.1016/j.cca.2017.11.005 29129476

[B42] Ye Y. Dai Q. Qi H. (2021). A Novel Defined Pyroptosis-Related Gene Signature for Predicting the Prognosis of Ovarian Cancer. Cell Death Discov. 7 (1), 71. 10.1038/s41420-021-00451-x 33828074 PMC8026591

[B43] Yegnasubramanian S. Kowalski J. Gonzalgo M. L. Zahurak M. Piantadosi S. Walsh P. C. (2004). Hypermethylation of CpG Islands in Primary and Metastatic Human Prostate Cancer. Cancer Res. 64 (6), 1975–1986. 10.1158/0008-5472.can-03-3972 15026333

[B44] Zeng C. Y. Li C. G. Shu J. X. Xu L. H. Ouyang D. Y. Mai F. Y. (2019). ATP Induces Caspase-3/gasdermin E-Mediated Pyroptosis in NLRP3 Pathway-Blocked Murine Macrophages. Apoptosis 24 (9-10), 703–717. 10.1007/s10495-019-01551-x 31175486

[B45] Zeng Y. Opeskin K. Goad J. Williams E. D. (2006). Tumor-induced Activation of Lymphatic Endothelial Cells via Vascular Endothelial Growth Factor Receptor-2 Is Critical for Prostate Cancer Lymphatic Metastasis. Cancer Res. 66 (19), 9566–9575. 10.1158/0008-5472.can-06-1488 17018613

[B46] Zhang J. S. Gong A. Young C. Y. (2007). ZNF185, an Actin-Cytoskeleton-Associated Growth Inhibitory LIM Protein in Prostate Cancer. Oncogene 26 (1), 111–122. 10.1038/sj.onc.1209769 16799630

[B47] Zhang Y. Tang L. (2017). Inhibition of Breast Cancer Cell Proliferation and Tumorigenesis by Long Non-coding RNA RPPH1 Down-Regulation of miR-122 Expression. Cancer Cell Int. 17, 109. 10.1186/s12935-017-0480-0 29200969 PMC5698957

[B48] Zhuang Z. Cai H. Lin H. Guan B. Wu Y. Zhang Y. (2021). Development and Validation of a Robust Pyroptosis-Related Signature for Predicting Prognosis and Immune Status in Patients with Colon Cancer. J. Oncol. 2021, 5818512. 10.1155/2021/5818512 34840571 PMC8616665

